# Doxycycline Resistance and 16S rRNA Mutations* in Treponema pallidum *(Response)

**DOI:** 10.3201/eid3208.260943

**Published:** 2026-08

**Authors:** George S. Long, Venkata R. Duvvuri

**Affiliations:** Public Health Ontario, Toronto, Ontario, Canada (G.S. Long, V.R. Duvvuri); University of Toronto, Toronto (V.R. Duvvuri)

**Keywords:** *Treponema pallidum*, syphilis, antimicrobial resistance, bacteria

**In Response:** On March 5, 2026, Drs. Lieberman and Beale contacted us concerning the identification of a heterozygous allele at position G966T (by *E. coli* reference numbering, G969T **by** *T. pallidum* reference numbering) of the 16S rRNA gene of *Treponema pallidum* subspecies *pallidum* (*1*). They surmised that our identification of the allele may have been caused by either genome misassemblies or contamination with sequences from other *Trepomena* species (*2*). In response to these reproducibility concerns, we undertook a separate analysis using an independent analytical process from our original work (*1*).

Our reanalysis focused on the sequencing libraries of Beale et al. (*3*–*5*) and applied a mapping-based approach, aligning reads to the 16S rRNA gene of the *T. pallidum* reference genome (NC_021490.2:231255–232803). That reanalysis concluded that the previously identified heterozygous allele was indeed the result of contamination. Accordingly, Table 4 and text associated with the heterozygous single-nucleotide polymorphism have been removed from the original article. In addition, an extra paragraph has been added to the Appendix detailing the additional analyses.

Of note, the overall findings of our study remain unaffected. We still report the sequencing and assembly of 15 *T. pallidum* subsp. *pallidum* genomes from Canada. This revision strengthens the clarity of our comparative genomics analysis and simplifies the identification of single-nucleotide polymorphisms related to putative doxycycline resistance and underscore the importance of accounting for the presence of two 16S rRNA gene copies in *T. pallidum.* This study continues to represent a vital step in establishing a genomic baseline for monitoring circulating *T. pallidum* subsp. *pallidum* lineages in Canada.

## References

[1] Long GS, Neale M, Braukmann T, Tran V, Singh N, Allen V, et al. Genomic analysis of doxycycline resistance–associated 16S rRNA mutations in *Treponema pallidum* subspecies *pallidum.* Emerg Infect Dis. 2026;32:242–5. 10.3201/eid3202.251060 41714842 PMC12928234

[2] Beale MA, Marks M, Luetkemeyer A, Celum C, Golden MR, Giacani L, et al. Doxycycline resistance and 16S rRNA mutations in *Treponema pallidum.* Emerg Infect Dis. 2026;32:1383–84. 10.3201/eid3208.260433 42521605 PMC13426860

[3] Beale MA, Marks M, Sahi SK, Tantalo LC, Nori AV, French P, et al. Genomic epidemiology of syphilis reveals independent emergence of macrolide resistance across multiple circulating lineages. Nat Commun. 2019;10:3255. 10.1038/s41467-019-11216-7 31332179 PMC6646400

[4] Beale MA, Marks M, Cole MJ, Lee MK, Pitt R, Ruis C, et al. Global phylogeny of *Treponema pallidum* lineages reveals recent expansion and spread of contemporary syphilis. Nat Microbiol. 2021;6:1549–60. 10.1038/s41564-021-01000-z 34819643 PMC8612932

[5] Beale MA, Thorn L, Cole MJ, Pitt R, Charles H, Ewens M, et al. Genomic epidemiology of syphilis in England: a population-based study. Lancet Microbe. 2023;4:e770–80. 10.1016/S2666-5247(23)00154-4 37722404 PMC10547597

